# Optogenetic demonstration of the involvement of SMA-negative mural cells in the regulation of cerebral blood flow

**DOI:** 10.3389/fphys.2023.1322250

**Published:** 2023-12-22

**Authors:** Chisato Iba, Yoshifumi Abe, Kenji F. Tanaka

**Affiliations:** Division of Brain Sciences, Institute for Advanced Medical Research, Keio University School of Medicine, Tokyo, Japan

**Keywords:** pericytes, venule smooth muscle cell, mural cell, vascular optogenetics, cerebral blood flow, tet-system

## Abstract

Mural cells are critical components of the cerebral vasculature. They are categorized into three primary subsets: arteriole smooth muscle cells (aSMCs), pericytes (PCs) and venule smooth muscle cells (vSMCs). It is well known that aSMCs can directly regulate cerebral blood flow (CBF) with their own contraction and dilation mechanisms. On the other hand, the direct involvement of PCs or vSMCs in CBF regulation is controversial. This ambiguity is largely due to the lack of specifically manipulable tools to isolate their function. To address this issue, we employed a set-subtraction approach by using a combination of tTA-mediated gene induction and Cre-mediated gene excision. We developed transgenic mice expressing optical actuators, channelrhodopsin-2 (ChR2) and photoactivated adenylyl cyclase (PAC) in smooth muscle actin (SMA)-negative mural cells that lack the machinery for SMA-mediated vasoregulation. Using these mouse models, we assessed CBF alterations in response to optical stimulation using laser Doppler techniques. Our results showed that optical stimulation induced notable CBF changes in both models. This study provides evidence for the potential regulatory role of PCs and vSMCs in cerebral hemodynamics and introduces powerful tools to specifically manipulate these cell types in vascular neurobiology.

## 1 Introduction

Mural cells surrounding arterioles, capillaries and venules are primarily classified into three types: arteriole smooth muscle cells (aSMCs), pericytes (PCs) and venule smooth muscle cells (vSMCs) (Vanlandewijck et al., 2018; Grant et al., 2019). aSMCs are typically defined as α-smooth muscle actin (SMA)-positive mural cells. SMA is one of the vital motor proteins for actomyosin-mediated vasoconstriction and vasodilation. Therefore, SMA-positive mural cells have the capacity to directly regulate local cerebral blood flow (CBF) through their contractile ability (Vanzetta et al., 2005; Hall et al., 2014; Kornfield and Newman, 2014; Kisler et al., 2017). On the other hand, vSMCs are SMA-negative mural cells (Vanlandewijck et al., 2018), suggesting that these cells lack the ability to directly constrict the vasculature.

The expression of SMA in PCs shows a gradual variance along the blood stream (Grant et al., 2019). Thus, ensheathing pericytes that are located around arterioles express SMA; thin-strand pericytes that are located on capillaries do not express SMA; mesh pericytes that are flanked by ensheathing and thin-strand pericytes also do not express SMA. Therefore, without classification of SMA-positive and SMA-negative PCs, it is difficult to address whether PCs directly regulate local CBF. Indeed, some studies demonstrated that PCs do not engage in CBF regulation (Hill et al., 2015; Wei et al., 2016), but other studies demonstrated the involvement of PCs in local CBF regulation (Shih and Hartmann, 2017; Hartmann et al., 2018).

Another hurdle in this research area is the absence of specific molecular markers for SMA-negative mural cells. *Pdgfrβ* and *Cspg4* (also known as neuron-glial antigen 2 (NG2)) have been commonly used to identify PCs. However, the transgenic animals that use these promoter activities induce a gene of interest in aSMCs as well. Indeed, recent single-cell RNA sequencing data have demonstrated that *Pdgfrβ* and *Cspg4* mRNA expression occurs in aSMCs as well as PCs and vSMCs (Vanlandewijck et al., 2018). These data imply that so called PC-specific promoters are active in all three types of mural cells. As a result, there are currently no systems to specifically induce a gene of interest in PCs and vSMCs.

Previous studies have shown that optogenetic stimulation of mural cells can alter local CBF (Mateo et al., 2017; Tran et al., 2018; Abe et al., 2021; Oishi et al., 2023). Optogenetic stimulation of channelrhodopsin (ChR2)-expressing aSMCs triggered vasoconstriction, resulting in a decrease in regional CBF (Hill et al., 2015). In contrast, the activation of photoactivated adenylyl cyclase (PAC), a protein that elevates intracellular cAMP levels upon exposure to blue light (Iseki et al., 2002; Yoshikawa et al., 2005; Zhou et al., 2016) induced vasodilation, leading to an increase in regional CBF (Abe et al., 2021). Direct dilation of blood vessels is known to be caused by an increase in intracellular cAMP in aSMCs (Lindsey et al., 2014). This experimental system, known as vascular optogenetics, can be used to explore the possible involvement of PCs and vSMCs in local CBF regulation (Nelson et al., 2020), provided optical actuators are specifically expressed in PCs and vSMCs. In this study, our aim was to develop new transgenic animal lines specifically expressing optical actuators (ChR2 or PAC) in SMA-negative mural cells and to investigate whether illumination of the optical actuator expressing SMA-negative mural cells could induce local CBF changes.

## 2 Methods

### 2.1 Animals

All animal procedures were conducted in accordance with the National Institutes of Health Guide for the Care and Use of Laboratory Animals and approved by the Animal Research Committee of Keio University School of Medicine (protocol number 18081). We used 17 male and 16 female mice (2–3 months old). The animals were maintained on a 12/12 h light/dark cycle in their home cage with free access to water and food.

The methods for generating *Cspg4*-tTA mice (NG2-tTA mice) (Tanaka et al., 2021), tetO*-*bPAC-2A-GFP mice (tetO-PAC mice) (Abe et al., 2021) and tetO-ChR2[C128S]-EYFP mice (tetO-ChR2 mice) (Tanaka et al., 2012) have been described previously. B6.FVB-Tg(Myh11-icre/ERT2)1Soff/J mice (Myh mice) (Jax strain ID: #019079) (Wirth et al., 2008) and B6.CgGt(ROSA)26Sortm14(CAG-tdTomato)Hze/J mice (Ai14 mice) (#007914) were obtained from the Jackson Laboratory. The following PCR primer sets were used for mouse genotyping: iCreU (5′- GAC AGA TGC CAG GAC ATC AG -3′) and iCreL (5′- ACC AGG CCA GGT ATC TCT G -3′) for Myh11-iCreERT2 mice, and MUT Fwd (5′- CTG TTC CTG TAC GGC ATG G -3′) and MUT Rev (5′- GGC ATT AAA GCA GCG TAT CC -3′) for Ai14 mice. The sizes of the PCR products were approximately 385, and 200 bp, respectively. Wild-type mice were negative for the above PCR products.

NG2-tTA::tetO-ChR2 double transgenic mice (hereafter NG2/ChR2 mice), NG2-tTA::tetO-ChR2; Myh11-iCreERT2 triple transgenic mice (NG2/ChR2/Myh mice), NG2-tTA::tetO-PAC double transgenic mice (NG2/PAC mice), NG2-tTA::tetO-PAC; Myh11-iCreERT2 triple transgenic mice (NG2/PAC/Myh mice), and NG2-tTA::tetO-PAC; Myh11-iCre ERT2; Ai14 quadruple transgenic mice (NG2/PAC/Myh/Ai14 mice) were used in this study.

NG2/ChR2/Myh mice, NG2/PAC/Myh mice and NG2/PAC/Myh/Ai mice were injected intraperitoneally with tamoxifen citrate (150 mg/kg, dissolved in corn oil, Tokyo Chemical Industry Co., LTD., Tokyo, Japan) daily from P21 to P25 to induce Cre-loxP recombination. Tamoxifen-injected mice were used for experiments approximately 1 month after the injection.

### 2.2 Immunohistochemistry

Detailed procedures were described in previous studies (Oishi et al., 2023). Briefly, mice were deeply anesthetized with an intraperitoneally administered mixture of ketamine-xylazine (100 mg/kg and 10 mg/kg, respectively). Perfusion was conducted via a cardiac route using 25 mL of glyoxal fixative (pH 5, containing 3% glyoxal, 20% ethanol, and 0.75% acetic acid). After perfusion, the brain was removed from the animals and post-fixed overnight in the same glyoxal fixative, and then stored in a 20% sucrose/phosphate buffered saline (PBS) solution. Sections (40 µm) were cut with a cryostat. The floating sections were permeabilized in PBS containing 0.1% TritonX-100 (PBS-T), and then incubated overnight with the following primary antibodies: goat anti-GFP polyclonal antibody (1:250, Rockland), mouse anti-SMA monoclonal antibody (clone 1A4, 1:1000, Santa Cruz Biotechnology) and rat anti-laminin α2 (laminin α2) monoclonal antibody (clone 4H8-2, 1:1000, Santa Cruz Biotechnology). On the following day, the sections were washed three times for 5 min with PBS-T, and then incubated with species-specific secondary antibodies conjugated to Alexa Fluor 488, 555, 649 for 2 h at room temperature. Then, the sections were washed three times for 5 min with PBS and mounted with medium composed of 97% 2,2′-Thiodiethanol (Sigma-Aldrich, 141 MO, United States) and 0.24% DABCO (Sigma-Aldrich, MO, United States) in PBS. Fluorescence images were obtained using a confocal laser scanning microscope (FLUOVIEW FV3000; Olympus, Tokyo, Japan). Images of primary sensory cortex captured with a ×20 objective lens were used for histological analysis.

### 2.3 Image analysis

The expression ratio of GFP in mural cells was analysed using ImageJ (https://imagej.nih.gov/ij/index.html) and the deep learning network system U-net (Falk et al., 2019). In a previous study, a new method was established to segment arterioles, capillaries, and venules by the results of SMA/laminin α2 immunohistochemical staining, and to measure the coverage ratio of GFP in the three vessel types (Oishi et al., 2023). The GFP expression ratio in the unilateral primary cortex was shown as the “GFP expression (%)” in each mouse. The total length of each mural cell type captured in the 5 images was used for quantitative analysis. Five representative images obtained with ×20 objective lenses from the unilateral primary sensory cortex were used to analyse tdTomato expression in Myh/Ai14 mice. TdTomato expression ratios were measured manually because their fluorescence in the soma tends to be too weak to be captured by a microscope.

### 2.4 Optogenetic stimulation and measurement of CBF using laser Doppler

Mice were anesthetized intraperitoneally with urethane (1.2 g/kg body weight), and placed in a head holder (SG-4N, modified to be flexible around the horizontal axis; Narishige Scientific Instrument Laboratory, Tokyo, Japan). All procedures were performed with the rectal temperature maintained at 37°C using a heating pad (BWT-100A; Bioresearch Center Co., Nagoya, Japan). The scalp was removed and blood was thoroughly removed if necessary. Laser Doppler (LDF; ALF 21, Advance Co., Ltd., Tokyo, Japan) probes (BF52; Advance Co., Ltd., Tokyo, Japan) joined to an optical stimulation fiber (400 nm in diameter) were placed on the cranial surface (1.1 mm anterior and 1.7 mm lateral from the bregma). During the experiments, continuous CBF was recorded using Power Lab (Power Lab8/30; AD Instruments, Ltd., Sydney, Australia). Optical stimulation was initiated at least 30 min after surgery.

For optogenetic stimulation, blue (465 nm) light was generated by 2-channel drivers (LEDD2; Doric Lenses, QC, Canada). Blue light illumination (0.3-, 1- and 3-s duration) (250 μW) was delivered from the tip of the optical fiber, and CBF responses were recorded by Doppler probes. The CBF responses were recorded for 150 s in each stimulation paradigm, with stimulation occurring 30 s after the onset of the recording. The intervals of each stimulation were at least 10 min. After all procedures, mice were perfused with glyoxal solution and the brains were used in immunohistochemical experiments as mentioned above.

### 2.5 Analysis of CBF

CBF data were analyzed using the MATLAB program (MathWorks, MA, United States) (Abe et al., 2021). The percentage change in CBF was calculated as ΔCBF(t) = (CBF(t)-CBF_pre_)/CBF_pre_ ×100, where CBF(t) is the Doppler signal at time t and CBF_pre_ is the Doppler signal averaged over 30 s before optogenetic stimulation. Peak change in CBF was defined as the maximum change in CBF. Tau on was defined as the time when the change in CBF reached 63.2% of change and tau off was defined as the time when the change in CBF reached 36.8% of the change after the peak.

### 2.6 Statistics

All analyses were performed using IBM SPSS Statistics 28 (Armonk, NY, United States). To assess differences in CBF change, a two-way ANOVA was employed for NG2/ChR2 mice and NG2/ChR2/Myh mice, as well as NG2/PAC mice and NG2/PAC/Myh mice. *p*-values are displayed with Bonferroni corrections. The paired *t*-test was used to evaluate differences in GFP expression between NG2/ChR2 mice and NG2/ChR2/Myh mice, and NG2/PAC mice and NG2/PAC/Myh mice. The results for CBF change and immunohistochemical staining are plotted in figures.

## 3 Results

### 3.1 Successful removal of optical actuators from SMA-positive mural cells

**FIGURE 1:**
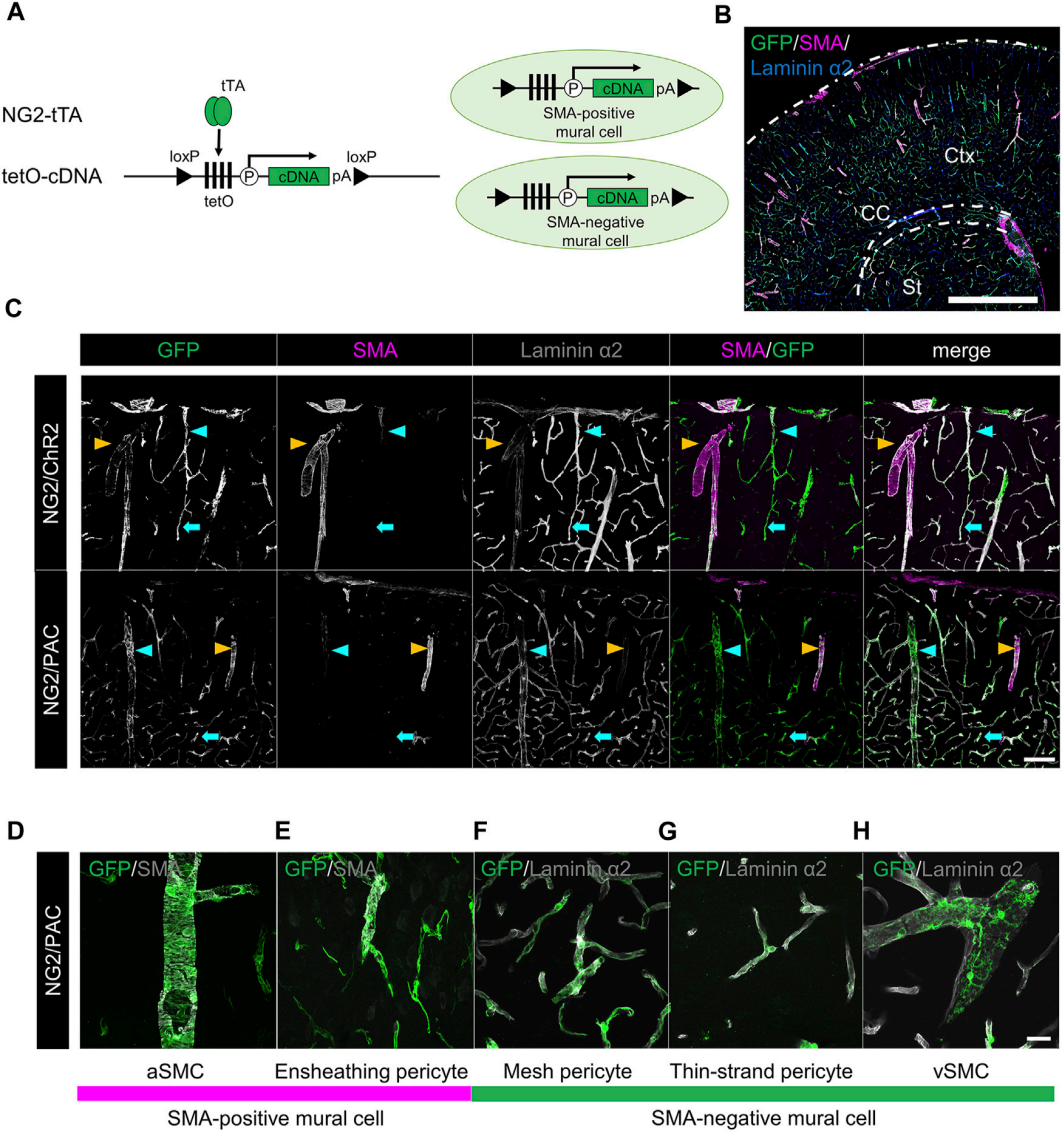
Establishment of transgenic animals expressing ChR2 or PAC in mural cells. **(A)** Diagrammatic summary of tTA-tetO system. NG2 promotor-driven expression of cDNA (ChR2 or PAC) in both SMA^+^ mural cells and SMA^−^ mural cells. tTA: tetracycline-controlled transcriptional activator; tetO: tetracycline operator; pA: polyadenylation signal; loxP: locus of X-over P1. **(B)** Representative confocal image at a macro level of GFP expression in NG2/ChR2 mice. GFP was expressed in all mural cells labeled by laminin α2 (blue). Ctx: cortex; CC: corpus callosum; St: striatum. Scale bar: 1 mm. **(C)** Representative confocal image at a micro level of immunohistochemistry with GFP, SMA, and laminin α2. In both NG2/ChR2 and NG2/PAC mice, GFP was expressed in all mural cells including SMA^+^ SMCs (yellow arrowheads), SMA^−^ PCs (cyan arrows) and SMA^−^ vSMCs (cyan arrowheads). SMA expression was observed in aSMCs, but not in PCs or vSMCs. Scale bar: 100 µm. **(D–H)** Representative images of each type of mural cell in NG2/PAC mice. **(D,E)** are classified as SMA-positive mural cells, **(F–H)** as SMA-negative mural cells. Scale bar: 25 µm.

NG2-tTA mice were crossed with tetO-ChR2 or tetO-PAC mice to generate NG2/ChR2 or NG2/PAC mice, respectively (Figure 1A). To confirm the gene induction pattern in mural cells of these mice, we performed triple immunohistochemical staining for GFP, SMA, and laminin α2 (Figure 1B). GFP antibody labeled actuator-expressing cells. Laminin α2 is a marker of the basal membrane so that whole cerebral blood vessels are delineated with laminin α2. We defined aSMCs as SMA^+^ cells associated with laminin α2^+^ blood vessels, and defined vSMCs as SMA^−^ cells associated with laminin α2^+^ blood vessels. PCs were further classified into three types by their SMA expression pattern and their morphology (Grant et al., 2019). Ensheathing PCs are continuums of aSMCs and surround pre-capillary arteriole circumferentially. Ensheathing PCs are commonly SMA-positive. Mesh PCs and thin-strand PCs attach to capillaries and are SMA-negative. GFP was expressed in all mural cells including aSMCs, vSMCs and three types of PCs in both NG2/ChR2 and NG2/PAC mice (Figures 1C–H) (Oishi et al., 2023).

**FIGURE 2:**
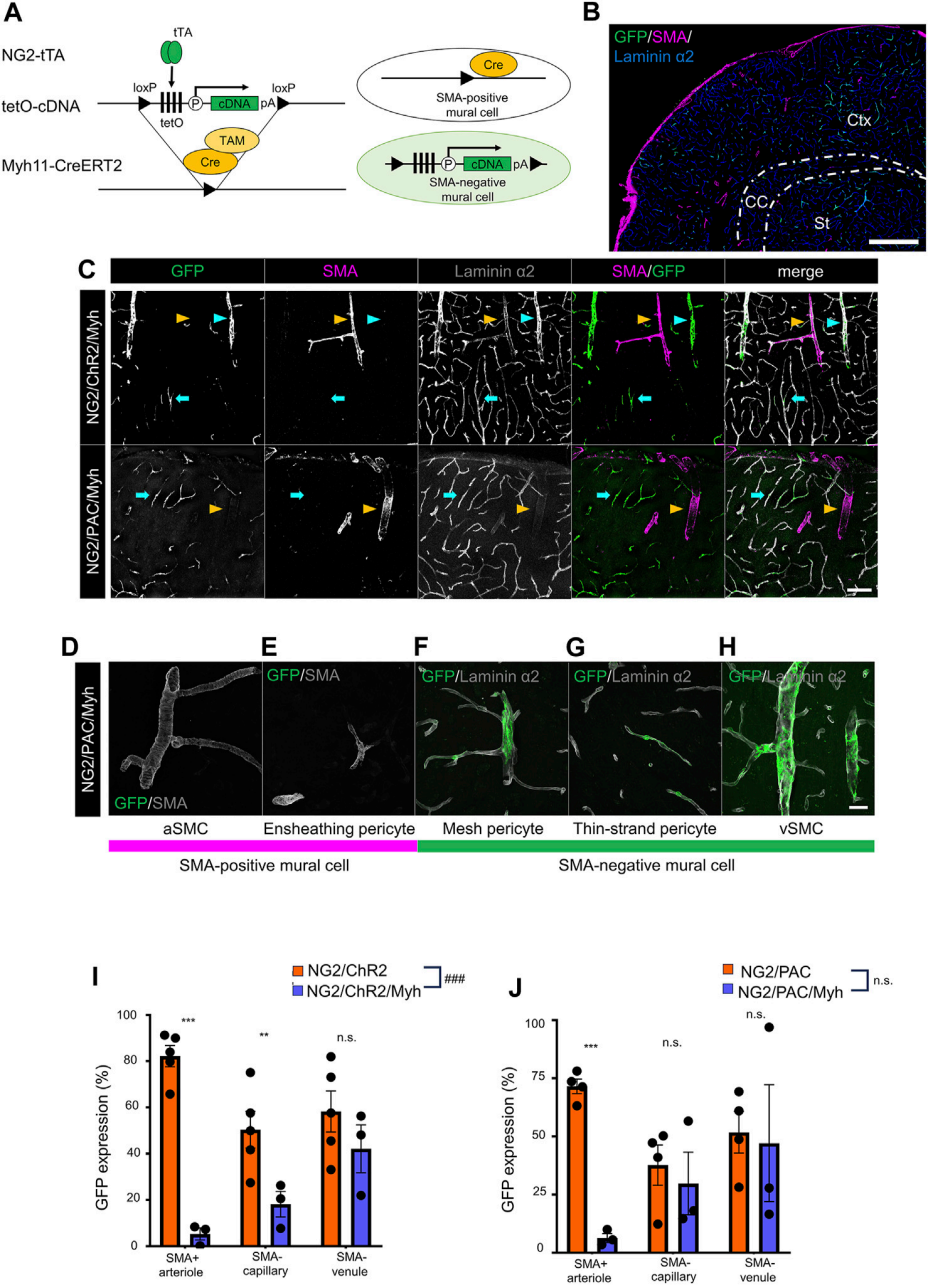
Establishment of a transgenic animal expressing ChR2 or PAC in SMA-negative mural cells. **(A)** Diagrammatic summary of the set-subtraction system that combines a tTA-tetO system and a Cre-loxP system. The tetO cassette is floxed by loxP. Under the presence of tamoxifen (TAM), the tetO cassette was removed by Cre recombination in SMA^+^ mural cells, thereby expressing the cDNA in SMA^−^ mural cells. tTA: tetracycline-controlled transcriptional activator; tetO: tetracycline operator; pA: polyadenylation signal; loxP: locus of X-over P1. **(B)** Representative image at a macro level of GFP expression in NG2/ChR2/Myh mice. GFP was expressed in SMA^−^ mural cells, but not in SMA^+^ mural cells. Scale bar: 500 µm. **(C)** Representative confocal image at a micro level of immunohistochemistry with GFP, SMA, and laminin α2. GFP was expressed in SMA^−^ PCs (cyan arrows) and SMA^−^ vSMCs (cyan arrowheads) in both NG2/ChR2/Myh mice and NG2/PAC/Myh mice. GFP^+^/SMA^+^ aSMCs (yellow arrowheads) are extremely rare in both mice. Scale bar: 100 µm. **(D–H)** Representative images of each type of mural cell in NG2/PAC/Myh mice. **(D,E)** are classified as SMA-positive mural cells, which do not express GFP. **(F–H)** are classified as SMA-negative mural cells and express GFP. Scale bar: 25 µm. **(I,J)** The GFP expression ratios were compared in SMA^+^ arterioles (*p* < 0.001 for NG2/ChR2/Myh, and NG2/PAC/Myh), in SMA^−^ capillaries (*p* = 0.016, 0.65), and SMA^−^ venules (*p* = 0.29, 0.87) between NG2/ChR2 (n = 5) and NG2/ChR2/Myh (n = 5) mice **(I)**, and NG2/PAC (n = 4) and NG2/PAC/Myh (n = 3) mice **(J)**. ***, *p* < 0.001; **, *p* < 0.01; *, *p* < 0.05 (Bonferroni’s corrected *p*-value). ###, *p* < 0.001 (two-way ANOVA; *p* < 0.001 for tetO-ChR2 line, *p* = 0.32 for tetO-PAC line). n.s.: not significant. All values are plotted. Bars show mean ± SEM.

We aimed to induce optic actuator expression specific to SMA-negative mural cells by using a set-subtraction strategy. This approach involves the excision of the loxP-flanked-tetO cassette in Cre recombinase-expressing cells, which abolishes tTA-mediated gene induction (Figure 2A). In this study, the *Myh11*-CreERT2 line was used to remove the tetO cassette from SMA-positive mural cells because *Myh11* is known as the gene corresponding to smooth muscle myosin heavy chain. After TAM administration, SMA-positive mural cells lost the ability of tTA-mediated gene induction, while SMA-negative mural cells retained tTA-mediated gene induction (Figure 2B).

We investigated whether actuator induction was limited in SMA-negative mural cells in NG2/ChR2/Myh and NG2/PAC/Myh mice after TAM administration. Thus, we performed the triple immunohistochemical staining for GFP, SMA, and laminin α2. In both NG2/ChR2/Myh and NG2/PAC/Myh mice, SMA-positive cells lost GFP immunoreactivities and SMA-negative cells retained them (Figures 2C–H), suggesting a removal of actuators from SMA-positive mural cells.

To quantify the degree of actuator induction in mural cells with or without Cre-mediated excision, we measured the GFP-labeling indices in the SMA-positive arteriole, the SMA-negative capillary and the SMA-negative venule. The GFP-labeling indices in NG2/ChR2 mice were 82.2% ± 4.6%, 50.4% ± 8.0% and 58.3% ± 8.9% in the SMA-positive arteriole, the SMA-negative capillary and the SMA-negative venule, respectively. In NG2/ChR2/Myh mice, the indices were 5.3% ± 2.6%, 18.2% ± 5.5% and 42.1% ± 10.3%, respectively (Figure 2I). Similarly, in NG2/PAC mice, the GFP-labeling indices were 71.6% ± 3.1%, 37.7% ± 8.6% and 51.8% ± 8.9%, respectively. In NG2/PAC/Myh mice, the indices were 6.4% ± 1.9%, 29.8% ± 13.4% and 47.1% ± 25.1%, respectively (Figure 2J). These data demonstrated a significant elimination of actuator induction from SMA-positive mural cells and a remaining actuator induction in SMA-negative mural cells through the set-subtraction strategy.

### 3.2 Establishing a unique transgenic mouse expressing GFP in *Myh11*-Cre-negative mural cells and tdTomato in *Myh11*-Cre-positive mural cells

**FIGURE 3:**
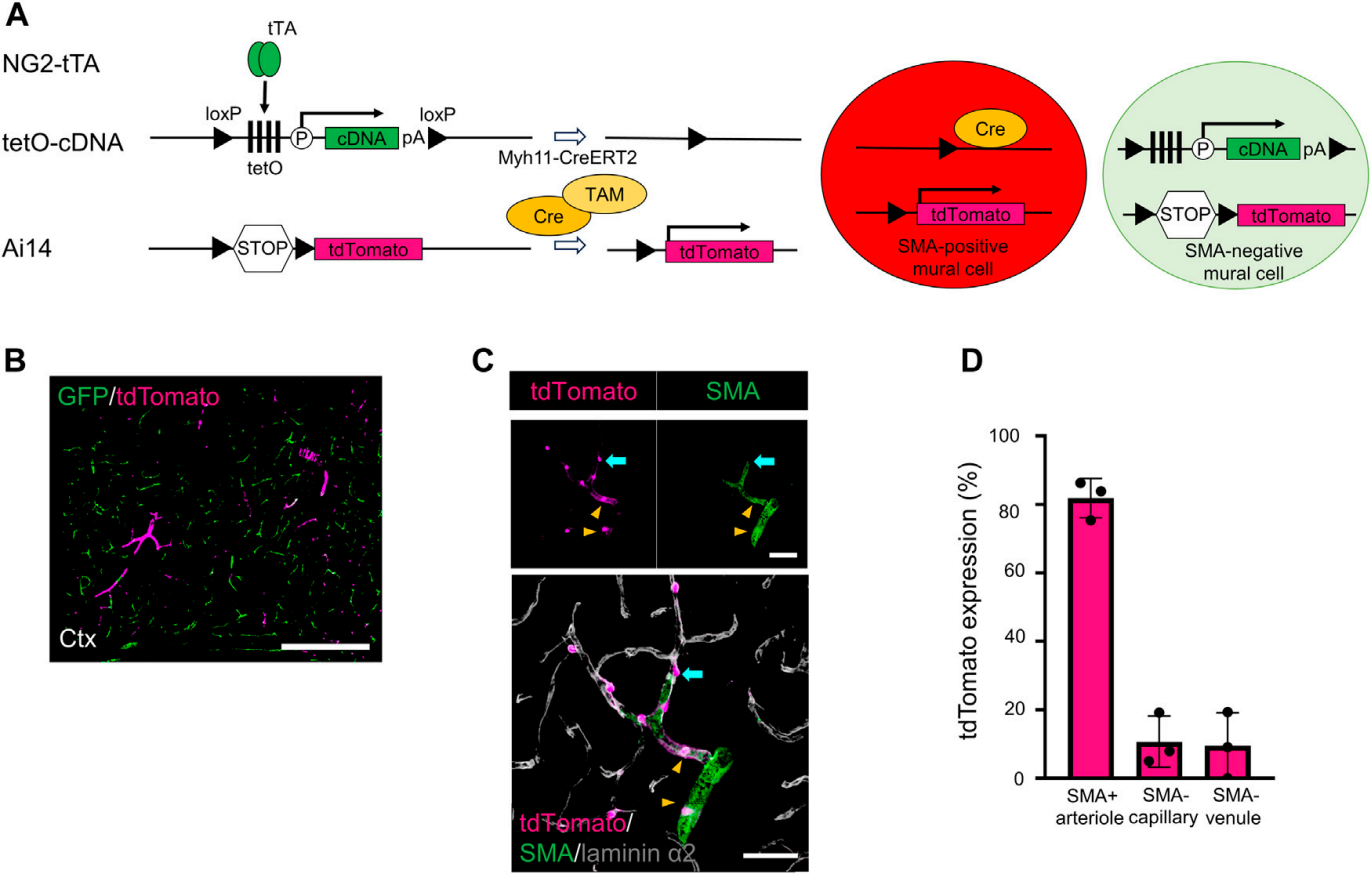
Generating transgenic mice expressing GFP in SMA-negative mural cells and tdTomato in SMA-positive mural cells. **(A)** Diagrammatic summary of the methods for creating a transgenic animal for this study. Under the presence of tamoxifen (TAM), Cre induces two types of induction in Myh11-positive cells: i) the floxed tetO cassette and the stop codon were removed in SMA^+^ mural cells, thereby expressing tdTomato in SMA^+^ mural cells and ii) the floxed tetO cassette and the stop codon were kept in SMA^−^ mural cells, thereby expressing GFP in SMA^−^ mural cells. tTA: tetracycline-controlled transcriptional activator; tetO: tetracycline operator; pA: polyadenylation signal; loxP: locus of X-over P1. **(B)** Representative confocal image at a macro level shows GFP (green) and tdTomato (magenta) expression in the cortex of NG2/PAC/Myh/Ai14 mice. GFP was expressed in SMA^−^ mural cells, and tdTomato was expressed in SMA^+^ mural cells. Scale bar: 500 µm. **(C)** Representative confocal image at a micro level shows tdTomato expression in SMA^+^ aSMCs (yellow arrowheads) and SMA^+^ PCs (cyan arrows). Grey shows laminin α2 in the merged image. Scale bars: 50 µm. **(D)** Quantitative analysis of tdTomato expression ratio in NG2/PAC/Myh/Ai14 mice in SMA^+^ arterioles, SMA^−^ capillaries and SMA^−^ venules (n = 3). All values are plotted. Bars show mean ± SEM.

We investigated why the GFP-labeling indices were slightly reduced in the SMA-negative capillaries and venules after Cre recombination. Recent single-cell RNA sequencing data demonstrated that some PCs and vSMCs weakly expressed *Myh11* mRNA (Vanlandewijck et al., 2018) (Supplementary Figure S1A). Consequently, weak *Myh11* gene expression could induce Cre expression. To visualize potential *Myh11* promoter activity, we established a unique transgenic mouse line (NG2/PAC/Myh/Ai14 mice) in which Cre-positive mural cells were labeled with tdTomato and Cre-negative mural cells were labeled with GFP (Figures 3A, B).

In this system, most SMA-positive mural cells expressed tdTomato (Figures 3B, D). In addition, some of the SMA-negative mural cells expressed tdTomato (Figures 3C, D), which reflected weak Cre activity and Myh11 expression in PCs and vSMCs (Vanlandewijck et al., 2018). These results explained why Myh11-Cre-mediated set-subtraction lowered the number of actuator-expressing cells among SMA-negative mural cells.

### 3.3 SMA-negative mural cells were capable of regulating local CBF

**FIGURE 4:**
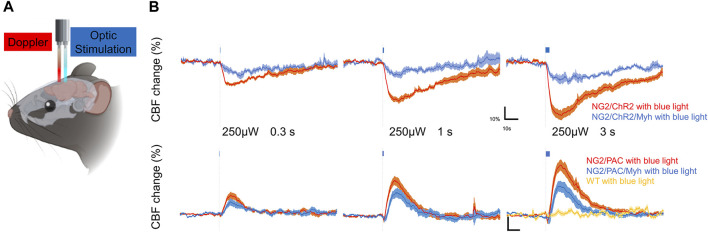
SMA-negative mural cells exhibited vascular optogenetic-induced CBF responses. **(A)** Diagrammatic summary of the experiment measuring vascular optogenetic-induced CBF. An optic fiber and a Doppler probe were placed on the skull surface of anesthetized mice. **(B)** Averaged CBF responses of NG2/ChR2 mice (*n* = 6), NG2/PAC mice (*n* = 9), NG2/ChR2/Myh mice (*n* = 4) and NG2/PAC/Myh mice (*n* = 8) upon blue light (465 nm) illumination (grey dotted line) lasting 0.3, 1 or 3 s. CBF responses in wild-type (WT) mice upon 3 s blue light illumination are shown in yellow line (*n* = 4).

To examine the potential of SMA-negative mural cells to regulate CBF, we optogenetically manipulated those cells over the skull and monitored CBF under anesthesia (Figure 4A). Blue light stimulation was administered with an intensity of 250 µW for stimulation durations of 0.3, 1, and 3 s. This stimulus paradigm has been demonstrated to be a reasonable intensity and duration to compare CBF changes when we used the ChR2(C128S) variant and PAC (Abe et al., 2021; Oishi et al., 2023). As expected, vascular optogenetic stimulation caused a decrease in CBF in NG2/ChR2 mice and an increase in CBF in NG2/PAC mice (Figure 4B). In both actuator expressing lines lacking Cre, the magnitudes of the optogenetic-induced CBF responses (trough and peak) were increased in a stimulation duration-dependent manner (Figure 4B; Table 1).

**TABLE 1:** Parameters in vascular optogenetic-induced CBF responses in each mouse. Comparison of trough (%), peak (%), time to trough (s), time to peak (s), tau on (s), and tau off (s) in CBF change between NG2/ChR2 mice (*n* = 6) and NG2/ChR2/Myh mice (*n* = 4 for most parameters. Exceptions as follows: *n* = 3 for tau off (1 s), time to trough (3 s), tau on (3 s) and tau off (3 s)), and NG2/PAC mice (*n* = 9 for most parameters, *n* = 7 for tau on (0.3 s), and *n* = 8 for tau on (1 s)) and NG2/PAC/Myh mice (*n* = 8 for most parameters, *n* = 5 for tau on (0.3 s), and *n* = 6 for tau on (1 s and 3 s)).

	Trough or peak (%)			Time to trough or peak(s)		
	0.3 s	1 s	3 s	0.3 s	1 s	3 s
NG2/ChR2	−22.3 ± 1.1	−34.8 ± 2.3	−50.2 ± 4.6	8.8 ± 0.8	12.5 ± 0.8	11.6 ± 0.4
NG2/ChR2/Myh	−14.9 ± 2.7	−13.7 ± 2.2	−20.3 ± 1.7	12.8 ± 2.3	11.1 ± 1.3	16.0 ± 1.3
	n.s.	***	**	n.s.	n.s.	*
ANOVA	###			#		
NG2/PAC	16.0 ± 2.5	28.4 ± 3.1	39.3 ± 4.6	9.9 ± 0.7	10.8 ± 0.6	13.2 ± 1.0
NG2/PAC/Myh	13.0 ± 3.4	20.2 ± 4.0	25.5 ± 5.3	10.6 ± 2.2	12.5 ± 0.9	12.2 ± 1.6
	n.s.	n.s.	n.s.	n.s.	n.s.	n.s.
ANOVA	n.s.			n.s.		

Tau on (s)			Tau off (s)		
0.3 s	1 s	3 s	0.3 s	1 s	3 s
3.4 ± 0.3	4.3 ± 0.3	3.8 ± 0.1	32.4 ± 0.3	32.7 ± 0.2	32.6 ± 0.1
7.2 ± 2.3	4.7 ± 1.0	4.3 ± 0.4	22.8 ± 6.2	28.6 ± 3.8	32.5 ± 0.7
n.s.	n.s.	n.s.	n.s.	n.s.	n.s.
#			#		
5.6 ± 0.4	5.3 ± 0.4	6.2 ± 0.3	17.8 ± 1.5	24.5 ± 2.7	27.7 ± 2.3
7.5 ± 1.1	7.3 ± 0.6	7.4 ± 0.7	17.9 ± 3.1	20.2 ± 2.1	22.0 ± 2.3
n.s.	*	n.s.	n.s.	n.s.	n.s.
n.s.			n.s.		

The values show means ± SEMs. #, *p* < 0.05; ##, *p* < 0.01; ###, *p* < 0.001 for two-way ANOVA test comparing NG2/ChR2 and NG2/ChR2/Myh mice, and NG2/PAC and NG2/PAC/Myh mice. *, *p* < 0.05; **, *p* < 0.01; ***, *p* < 0.001 for Student’s t-test comparing duration of NG2/ChR2 and NG2/ChR2/Myh mice, and NG2/PAC and NG2/PAC/Myh mice. Bonferroni correction is applied to adjust *p*-value. n.s.: not significant.

In actuator expressing lines with Cre, the illumination induced a clear response in CBF (Figure 4B). However, the trough of the CBF reduction (trough) in NG2/ChR2/Myh mice was significantly lower than that in NG2/ChR2 mice (Table 1). The times to trough and tau on in NG2/ChR2/Myh mice were significantly longer, and tau off was significant shorter than that in NG2/ChR2 mice. On the other hand, there were no differences in the peak of CBF change between NG2/PAC mice and NG2/PAC/Myh mice. All parameters in NG2/PAC/Myh mice were comparable with NG2/PAC mice, except tau on. Temporal parameters of CBF changes in each mouse type are outlined in Table 1.

To examine whether light stimulus artifacts (e.g., heat generated by the light) affected CBF changes (Rungta et al., 2017), 3 s blue light illumination was applied to wild-type mice. We found that blue light illumination did not cause CBF changes in wild-type mice (Figure 4B), indicating that the vascular optogenetic-induced CBF responses upon blue light illumination in NG2/ChR2/Myh and NG2/PAC/Myh mice were unlikely to be due to heat artefacts.

## 4 Discussion

In this study, we successfully generated two transgenic mouse models (NG2/ChR2/Myh and NG2/PAC/Myh) that predominantly expressed the optical actuators ChR2 and PAC in SMA-negative mural cells. Illumination over the skull induced CBF changes in both models, indicating the ability to regulate CBF in these cells. Here, we discuss probable mechanisms that induce CBF changes after optogenetic stimulation of both models.

### 4.1 Residual expression of ChR2 and PAC in SMA-positive cells directly affects optogenetic-mediated CBF changes

We employed a set-subtraction system that combined the tTA-tetO system and the Cre-loxP system to remove ChR2 or PAC expression from SMA-positive mural cells. We used *Myh11*-CreERT2 to remove optical actuators. GFP immunoreactivity remained sparse at 5.3% ± 2.6% of SMA-positive mural cells in NG2/ChR2/Myh mice, and 6.4% ± 1.9% in NG2/PAC/Myh mice after TAM administration. It is possible that GFP-negative cells express residual actuators after Cre-mediated removal and that these actuators alter CBF changes. However, we know empirically that the residual actuators, whose levels are undetectable by GFP immunohistochemistry, are unlikely to alter CBF changes. In contrast, it is possible that the actuator expression in 5%–6% of SMA-positive mural cells was sufficient to induce CBF changes. In particular, it is likely that aSMCs, sparsely labeled by ChR2, constrict upon illumination and decreased CBF by the bottle neck effect. Sparse and specific actuator induction in aSMCs will be necessary to address this point.

### 4.2 The contractile ability of SMA-negative PCs and vSMCs themselves caused the optogenetic-mediated CBF changes

While actomyosin-mediated contractility is a widely conserved mechanism in aSMCs to regulate CBF, we wondered why SMA-negative cells were able to contract or dilate without the actomyosin complex. Actin protein has at least six subtypes in mammals: α_skeletal_-encoded by *Acta1*, α_cardiac_-Actin encoded by *Actc1,* α_smooth_-Actin encoded by *Acta2* which is referred as SMA in this paper, β_cyto_-Actin encoded by *Actb*, γ_cyto_-Actin encoded by *Actg1*, and γ_smooth_-Actin encoded by *Actg2* (Perrin and Ervasti, 2010)*.* Among these subtypes, actin encoded by *Acta2* and *Actg2* are involved in actomyosin-mediated vasoconstriction and vasodilation, and they are highly expressed in aSMCs and merely expressed in PCs and vSMCs (Vanlandewijck et al., 2018). Therefore, we initially hypothesized that SMA-negative mural cells are unlikely to contract and dilate on their own.

Surprisingly, several groups demonstrated that PCs and/or vSMCs had contractile ability. Hartmann et al. utilized two-photon microscopy and observed a reduction in capillary diameter following local optogenetic activation of ChR2-positive, SMA-negative mural cells (Hartmann et al., 2018). Murata et al. also showed a decrease in capillary diameters after local optogenetic stimulation of ChR2 on PCs but not vSMCs (Murata et al., 2023). However, these studies did not completely exclud the possibility that SMA-positive PCs were optogenetically targeted. On the other hand, Hill et al. did not observe changes in capillary diameters following local two-photon stimulation of ChR2 in SMA-negative mural cells (Hill et al., 2015), supporting the notion that SMA-negative PCs and vSMCs had no contractile ability.

Our findings indicated that SMA-negative PCs and vSMCs somehow regulate CBF, however, we did not directly observe the motility of mural cells and additional evidence is required to arrive at a definitive conclusion.

### 4.3 The possibility that SMA-negative PCs and vSMCs indirectly affected aSMC function and altered CBF

The third possible mechanism of optogenetic-mediated CBF changes is that aSMC contractility is indirectly affected by adjacent SMA-negative PCs and vSMCs. For example, when PCs and vSMCs depolarize, they release potassium ions, which could depolarize adjacent SMA-positive SMCs, resulting in contraction of blood vessels (Kisler et al., 2017). Further investigations are needed to identify secreted bioactive molecules from SMA-negative PCs or vSMCs after optogenetic stimulation. The system that enables us to easily control extracellular conditions, such as acute brain slice (Fernández-Klett et al., 2010) and whole-mount retina (Alarcon-Martinez et al., 2019), would be ideal to prove aSMC-modulatory factors from SMA-negative mural cells.

### 4.4 Possible false-negative for the SMA immunoreactivity

A previous study reported that SMA immunoreactivity can vary between tissue preparations; the SMA immunoreactivity decreases massively after PFA fixation, and is maintained by actin stabilization followed by methanol fixation (Alarcon-Martinez et al., 2018). In this highly sensitive condition, the SMA immunoreactivity was detected in some of the retinal capillary pericytes (Alarcon-Martinez et al., 2018). In our experiments, we exploited a glyoxal perfusion fixation and obtained the greater SMA immunoreactivity than PFA perfusion fixation, and classified SMA-positive and negative mural cells based on these results. However, if the sensitivity of SMA detection in our methods was not maximized, the quantification of the number of SMA-positive cells would be discounted and the classification of the population we have performed might change. We must acknowledge the possibility of variation of SMA immunoreactivity as a limitation of this study.

## Data Availability

The raw data supporting the conclusion of this article will be made available by the authors, without undue reservation.
