# Supplementary material for: Optogenetic demonstration of the involvement of SMA-negative mural cells in the regulation of cerebral blood flow
Source: Front Physiol. 2023 Dec 22;14:1322250. doi: 10.3389/fphys.2023.1322250 (PMC10771846; doi:10.3389/fphys.2023.1322250)
Supplement: Supplementary file 1 [file DataSheet1.docx]

Supplementary Material

## Supplementary Figure


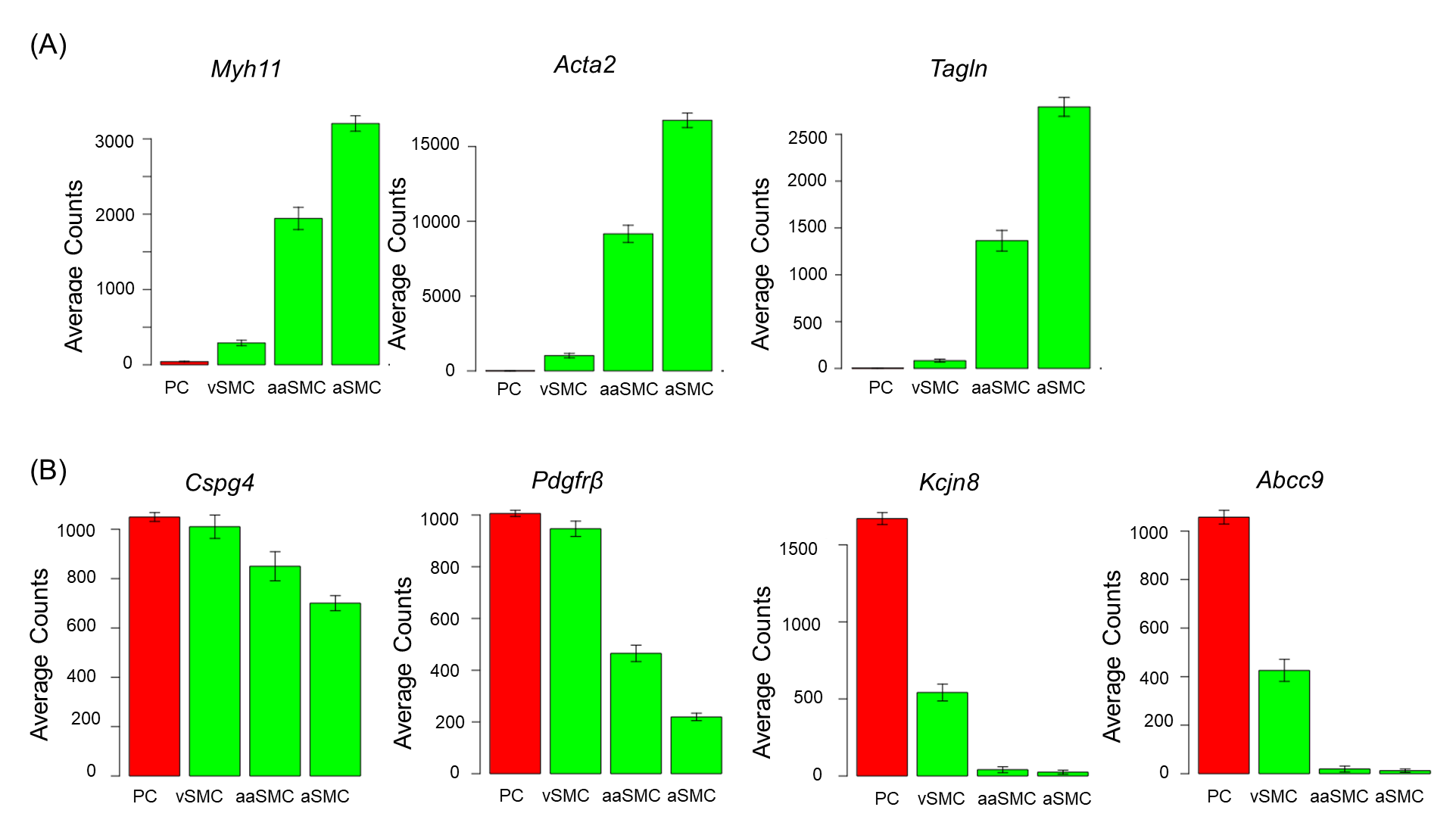


**Supplementary Figure 1. Marker mRNA expression in each type of mural cell**

Single-cell RNA sequence data revealed that *Pdgfrβ* and *Cspg4* mRNA were expressed in PC, vSMC, aaSMC and aSMC. *Abcc9* and *Kcjn8* mRNA were expressed in PC and vSMC but were only slightly expressed in aaSMC, and aSMC (Vanlandewijck et al., 2018). PC: pericyte; vSMC: venule smooth muscle; aaSMC: arteriolar SMC; aSMC: arteriole SMC.
